# Digging for biosynthetic dark matter

**DOI:** 10.7554/eLife.06453

**Published:** 2015-02-13

**Authors:** Jia Jia Zhang, Bradley S Moore

**Affiliations:** Center for Marine Biotechnology and Biomedicine, Scripps Institution of Oceanography, University of California, San Diego, San Diego, United States; Skaggs School of Pharmacy and Pharmaceutical Sciences and the Scripps Institution of Oceanography, University of California, San Diego, San Diego, United Statesbsmoore@ucsd.edu

**Keywords:** metagenomics, natural products, biosynthesis, biogeography, None

## Abstract

An analysis of bacterial communities in soil samples from around the world reveals unexplored diversity in biosynthetic enzymes.

**Related research article** Charlop-Powers Z, Owen JG, Reddy BV, Ternei MA, Guimaraes DO, de Frias UA, Pupo MT, Seepe P, Feng Z, Brady SF. 2015. Global biogeographic sampling of bacterial secondary metabolism. *eLife* **4**:e05048. doi: 10.7554/eLife.05048

**Image** Soil samples were collected from five continents as part of a citizen-science project called ‘Drugs from Dirt’

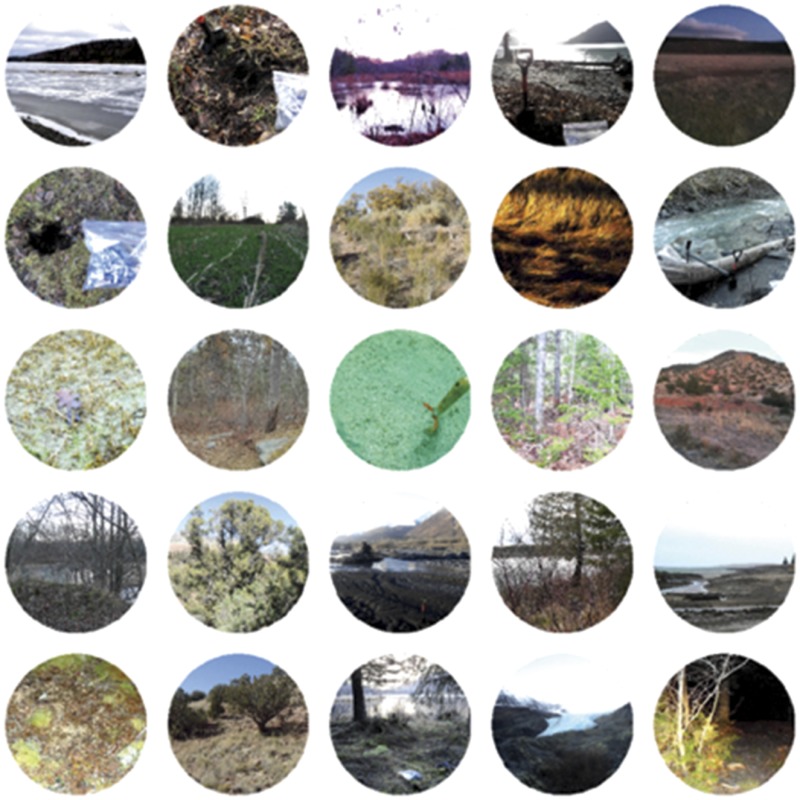

Researchers have long been interested in natural products—small molecules produced by an organism that are not directly involved in its normal growth, development or reproduction—as potential new medicines. However, despite success in the past, most pharmaceutical companies have abandoned natural product drug discovery efforts over the last decade because it has been difficult to reliably access a sufficient supply of these compounds. Moreover, efforts to find new bioactive chemicals have frequently ‘re-discovered’ the same chemical compounds (Li and Vederas, 2009).

Recently, research in the field has been revitalized by the realization that most natural products are encoded by clustered sets of genes that can be identified from genome sequence data of bacteria or bacterial communities. New findings suggest the biosynthetic potential of microbes actually remains largely untapped, as many ‘talented producers’ of small molecules cannot currently be cultured in the lab (Wilson et al., 2014). Furthermore, even bacteria that do grow in the lab possess more biosynthetic gene clusters than the number of natural products experimentally isolated from them (Cimermancic et al., 2014). For the first time, modern high-throughput sequencing technologies provide a means for sensing previously undetected ‘biosynthetic dark matter’.

Now, in *eLife*, Sean Brady of Rockefeller University and colleagues from the US, Brazil, South Africa and China—including Zachary Charlop-Powers as first author—leverage this knowledge to explore biosynthetic diversity from across the globe (Figure 1). As part of a citizen science project called ‘Drugs from Dirt’, the public was invited to collect and submit soil samples from around the world. Combining these with other soil samples gave a total of 96 samples from five continents and several oceanic islands. Charlop-Powers et al. then amplified, by PCR, conserved regions of genes for two enzymes involved in producing many natural products (namely non-ribosomal peptide synthetase and polyketide synthase). Next, they undertook a large-scale sequencing effort to identify and compare these two biosynthetic genes from this sampling of soil-dwelling bacteria (Charlop-Powers et al., 2015).

**Figure 1.**
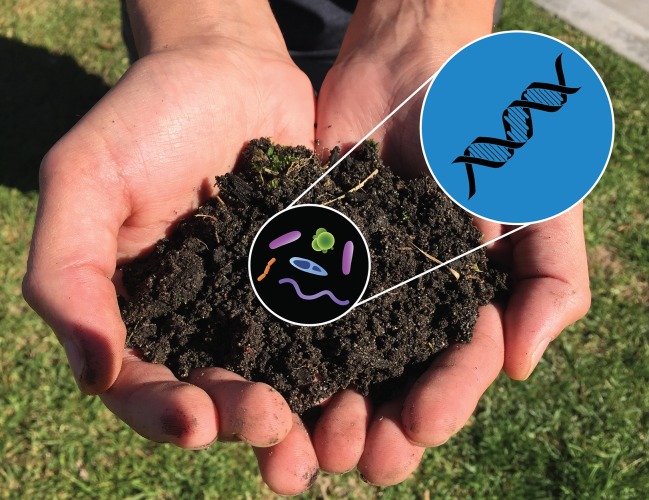
Unearthing biosynthetic diversity in soil-dwelling bacteria. Charlop-Powers et al. undertook a large-scale PCR and sequencing effort to identify and compare biosynthetic genes from bacterial communities in soil samples collected from around the world.

FIGURE CREDIT: PHOTOGRAPH BY ABRAHIM EL GAMAL.

The researchers found that the geographical location was the single most important factor that determined the sequence composition of the two biosynthetic genes. By systematically comparing sequence compositions to identify those that were most similar in the samples studied, Charlop-Powers et al. found that the strongest sequence overlap belonged to samples collected in close physical proximity to one another. Very little overlap was observed between samples collected from different global locations (less than 3%), even if they were collected from similar biomes (that is, regions with similar climates and ecosystems).

At the same time, biosynthetic sequence composition was influenced by biome-type on a more local level. When the researchers compared seven samples collected in New Mexico—one from a hot spring and six from arid topsoil biomes—they found that sequences from the dry soil samples were more similar to each other than to the sample collected from the hot spring, even though some dry sampling sites were physically closer to the hot spring than to each other. Additionally, samples collected from different sites spanning three Brazilian biomes (aquatic, forest, and tropical savanna) were most strongly related to samples collected from the same Brazilian biome and only distantly related to samples from other biomes, regardless of how close they were geographically.

Upon reflection, the findings from this small sampling of biosynthetic diversity are not so surprising. Previous studies have demonstrated convincing evidence that biosynthetic genes and gene clusters are frequently exchanged by horizontal gene transfer (Ziemert et al., 2014), and microbes can only share genetic material in this manner if they can physically interact. The fact that biome-type also has an influence on sequence composition indicates that natural products may play significant roles in microbial ecology.

What are those roles? For the most part, we have no idea. The ability to make natural products is clearly important, as many well-studied soil bacteria devote significant portions of their genomes to the production of natural products, in some cases up to 10% (Udwary et al., 2007). It has long been assumed that these compounds are defensive, as many natural products have antibiotic activity and confer fitness benefits to producers competing for nutrients (Kinkel et al., 2014). However, doses of these compounds that are too low to inhibit or kill other microbes can still alter their gene expression, and this scenario may be more relevant in the environment (Goh et al., 2002). Given the vast diversity of biosynthetic potential uncovered in this survey, are the functions and impacts of natural products in the environment equally varied?

Although the work of Brady, Charlop-Powers and co-workers cannot offer direct answers to these questions, it does offer a more tangible contribution to drug discovery efforts. By using known sequences from characterized gene clusters, the researchers identified samples enriched with biosynthetic genes similar to those associated with biomedically relevant compounds. These biosynthetic ‘hot spots’ may serve as starting points to search for structurally similar molecules with, hopefully, equally potent activities. The challenge now will be to work out how to translate sequence information into chemical reality, and further, how to begin to access not just small variations in old chemistry, but completely new chemical scaffolds.
